# MicroRNA-665-3p exacerbates nonalcoholic fatty liver disease in mice

**DOI:** 10.1080/21655979.2021.2017698

**Published:** 2022-01-18

**Authors:** Yuanjie Yu, Tian Tian, Shiyun Tan, Pengbo Wu, Yitian Guo, Ming Li, Mengjun Huang

**Affiliations:** a Department of Gastroenterology, Renmin Hospital of Wuhan University, Wuhan, China; b Hubei Key Laboratory of Digestive System Disease, Renmin Hospital of Wuhan University, Wuhan, China; c Department of Nutrition, The Central Hospital of Wuhan, Tongji Medical College, Huazhong University of Science and Technology, Wuhan, China

**Keywords:** Mir-665-3p, NAFLD, oxidative stress, inflammation, AMPKα, FNDC5

## Abstract

Oxidative stress and chronic inflammation are major culprits of nonalcoholic fatty liver disease (NAFLD). MicroRNA-665-3p (miR-665-3p) is implicated in regulating inflammation and oxidative stress; however, its role and molecular basis in NAFLD remain elusive. Herein, we measured a significant upregulation of miR-665-3p level in the liver and primary hepatocytes upon high fat diet (HFD) or 0.5 mmol/L palmitic acid plus 1.0 mmol/L oleic acid stimulation, and the elevated miR-665-3p expression aggravated oxidative stress, inflammation and NAFLD progression in mice. In contrast, miR-665-3p inhibition by the miR-665-3p antagomir significantly prevented HFD-induced oxidative stress, inflammation and hepatic dysfunction in vivo. Manipulation of miR-665-3p in primary hepatocytes also caused similar phenotypic alterations in vitro. Mechanistically, we demonstrated that miR-665-3p directly bound to the 3ʹ-untranslated region of fibronectin type III domain-containing 5 (FNDC5) to downregulate its expression and inactivated the downstream AMP-activated protein kinase alpha (AMPKα) pathway, thereby facilitating oxidative stress, inflammation and NAFLD progression. Our findings identify miR-665-3p as an endogenous positive regulator of NAFLD via inactivating FNDC5/AMPKα pathway, and inhibiting miR-665-3p may provide novel therapeutic strategies to treat NAFLD.

## 1. Introduction

Nonalcoholic fatty liver disease (NAFLD) has becoming one of the most common chronic liver diseases worldwide due to the increased prevalence of metabolic syndrome, and causes great personal suffering and socioeconomic burden [1–4]. Emerging studies have identified oxidative stress and chronic inflammation as major culprits of NAFLD, and inhibiting reactive oxygen species (ROS) overproduction and hepatic inflammation effectively ameliorates hepatocyte injury, fibrosis and hepatic dysfunction [5–9]. AMP-activated protein kinase alpha (AMPKα) is a central regulator of energy metabolism and serves as a promising therapeutic target to treat metabolic diseases [10–12]. In addition, AMPKα is also involved in controlling redox homeostasis and inflammatory response. It has been reported that AMPKα activation directly induces the expression of various antioxidant enzymes and reduces the generation of free radicals [13–15]. And AMPKα activation also suppresses the phosphorylation and nuclear translocation of nuclear factor kappa B (NF-κB), thereby preventing the transcription of downstream proinflammatory cytokines, including interleukin-1 beta (IL-1β), IL-6, monocyte chemotactic protein-1 (MCP-1) and tumor necrosis factor-alpha (TNF-α) [16]. As expected, Garcia et al demonstrated that hepatic steatosis and inflammation in high fat diet (HFD)-stimulated mice were dramatically attenuated by genetic liver-specific AMPKα activation [17]. Therefore, it is reasonable to treat NAFLD via activating AMPKα.

The discovery of endogenous noncoding RNAs helps to establish a new field to investigate the mechanism of NAFLD progression. MicroRNAs (miRNAs) are a class of single-stranded, small noncoding RNAs that negatively regulate gene expression at the posttranscriptional level via binding to the 3ʹ-untranslated region (UTR) of target mRNAs [18–22]. Notably, the expressions of various miRNAs have been found to be dysregulated in NAFLD patients [23]. Recent studies have identified a critical role of miR-665-3p in regulating inflammation and oxidative stress. Li et al found that inhibition of miR-665-3p significantly suppressed systemic inflammation in intestinal ischemia/reperfusion (I/R) injury, and Guo et al also demonstrated an inhibitory role of the miR-665-3p antagomir on inflammation in fusarium solani-associated keratitis [24,25]. In contrast, Zhang et al showed that miR-665-3p overexpression blocked the activation of NF-κB signaling in microglial cells upon oxygen-glucose deprivation stimulation [26]. In addition, miR-665-3p expression was found to be upregulated in rat failing hearts, and miR-665-3p inhibition significantly reduced cardiac oxidative stress and inflammation [27]. And miR-665-3p downregulation by dexmedetomidine conferred protective effects on oxidative stress and I/R-induced cardiac injury [28]. Based on these findings, we herein aim to investigate the role of miR-665-3p in oxidative stress, inflammation and NAFLD progression by in vivo and in vitro studies, and elucidate whether these effects are mediated by AMPKα.

## 2. Materials and methods

### 2.1 Reagents

The miR-665-3p agomir (#miR40003733-4-5), agomir control (#miR4N0000001-4-5), miR-665-3p antagomir (#miR30003733-4-5) and antagomir control (#miR3N0000001-4-5) were purchased from Guangzhou RiboBio Co., Ltd (Guangzhou, China). Compound C (CpC, #171,260) and 2ʹ,7ʹ-dichlorodi-hydrofluorescein diacetate (DCFH-DA, #D6883) were purchased from Sigma-Aldrich (St. Louis, MO, USA). Short hairpin RNAs against FNDC5 (shFNDC5) and scramble shRNA were purchased from Santa Cruz Biotechnology (Dallas, Texas, USA), and then packaged into the liver-specific adeno-associated virus serotype 8 (AAV8) vectors by Shanghai GenePharma Co.,Ltd. (Shanghai, China). RIPA lysis buffer (#G2002) and RT First Strand cDNA Synthesis Kit (#G3330) were purchased from Servicebio (Wuhan, China). Pierce™ BCA Protein Assay kit (#23,225), TRIzol™ Reagent (#15,596,018) and insulin Mouse ELISA Kit (#EMINS) were purchased from Thermo Fisher Scientific (Waltham, MA, USA). Lipofectamine 6000^TM^ reagent (#C0526), hydrogen peroxide (H_2_O_2_) assay kit (#S0038) and malondialdehyde (MDA) assay kit (#S0131S) were purchased from Beyotime (Shanghai, China). The assay kits to detect triglyceride (TG, #290-63,701), total cholesterol (TC, #294-65,801) and nonestesterified fatty acid (NEFA, #294-63,601) were purchased from Wako (Osaka, Japan). Mouse IL-1β ELISA Kit (#ab197742), mouse IL-6 ELISA Kit (#ab222503), mouse MCP-1 ELISA Kit (#ab208979), mouse TNF-α ELISA Kit (#ab208348), mouse IL-10 ELISA Kit (#ab108870) and lactate dehydrogenase (LDH) Assay Kit (#ab102526) were purchased from Abcam (Cambridge, UK). Irisin ELISA Kit (#SK00170-01) was purchased from Aviscera Bioscience (Santa Clara, CA, USA). The primary antibodies against phospho-AMPKα (p-AMPKα, #50,081) and total-AMPKα (t-AMPKα, #5832) were purchased from Cell Signaling Technology (Beverly, MA, USA). Anti-fibronectin type III domain-containing 5 (FNDC5, #ab174833) and anti-β-actin (#ab8226) were purchased from Abcam.

### 2.2 Animals and treatments

C57BL/6 male mice aged 8–10 weeks were fed with a HFD (60% kcal fat, 20% kcal carbohydrates and 20% kcal protein) for 24 weeks to establish NAFLD model as previously described, and the matched control mice were fed with a normal diet (ND, 10% kcal fat, 70% kcal carbohydrates and 20% kcal protein) for 24 weeks [29]. To manipulate hepatic miR-665-3p levels, mice were intraperitoneally injected with the miR-665-3p agomir, miR-665-3p antagomir or matched negative controls at a dose of 100 mg/kg weekly for 6 consecutive weeks before the mice sacrificed. To inhibit AMPKα, mice were intraperitoneally injected with CpC (20 mg/kg) once two days for 8 consecutive weeks before the mice sacrificed [30]. To knock down hepatic FNDC5, mice received a single intravenous injection of shFNDC5 from the tail vein at a concentration of 1 × 10^11^ viral genome per mouse for 4 weeks before the miR-665-3p antagomir treatment [31,32]. All animal procedures were approved by our institution’s ethical guidelines and also in compliance with the *Animal Research: Reporting of In Vivo Experiments* guidelines.

### 2.3 Western blot

Total proteins were extracted from the livers or primary hepatocytes using RIPA lysis buffer, and the concentrations were quantified using Pierce™ BCA Protein Assay kit [33–36]. Then, 20 μg total proteins were separated by 10% sodium dodecyl sulfate-polyacrylamide gel electrophoresis, transferred onto polyvinylidene fluoride membranes and incubated with the primary antibodies at 4°C overnight after being blocked with 5% skimmed milk at room temperature for 1 h. On the next day, the membranes were incubated with the horseradish peroxidase-conjugated secondary antibodies at room temperature for an additional 1 h, and then visualized by LumiGLO chemiluminescent substrate. β-actin was used as the house-keeping gene and the data were analyzed using an Image Lab software.

### 2.4 Quantitative real-time PCR

Total RNA was extracted using TRIzol™ Reagent and then subjected to the reverse transcription using a RT First Strand cDNA Synthesis Kit according to the manufacturer’s instructions. Quantitative real-time PCR was performed using a SYBR Green Master Mix, and gene expression was calculated using β-actin or U6 as the reference [37–40]. The primers were synthetized by Sangon Biotech (Shanghai) Co., Ltd. (Shanghai, China), and listed as below: collagen 1 alpha 1 (Col1α1): forward, 5ʹ-TGCTAACGTGGTTCGTGACCGT-3ʹ, reverse, 5ʹ-ACATCTTGAGGTCGCGGCATGT-3ʹ; Col3α1: forward, 5ʹ-ACGTAAGCACTGGTGGACAG-3ʹ, reverse, 5ʹ-CCGGCTGGAAAGAAGTCTGA-3ʹ; connective tissue growth factor (CTGF): forward, 5ʹ-TGACCCCTGCGACCCACA-3ʹ, reverse, 5ʹ-TACACCGACCCACCGAAGACACAG-3ʹ; transforming growth factor-beta 1 (TGF-β1): forward, 5ʹ-ATTTGGAGCCTGGACACACA-3ʹ, reverse, 5ʹ-GAGCGCACAATCATGTTGGA-3ʹ; FNDC5; forward, 5ʹ-ATGAAGGAGATGGGGAGGAA-3ʹ, reverse, 5ʹ-GCGGCAGAAGAGAGCTATAACA-3ʹ and β-actin: forward, 5ʹ-GTGACGTTGACATCCGTAAAGA-3ʹ, reverse, 5ʹ-GCCGGACTCATCGTACTCC-3ʹ.

### 2.5 Histologic analysis

The livers were quickly excised and fixed in 4% (w/v) paraformaldehyde, embedded in paraffin and sectioned to 5 μm slices. Next, paraffin-embedded sections were exposed to dewaxing, hydratization and hematoxylin and eosin (HE) staining by standard procedures. Oil red O staining was performed on frozen tissue sections to measure lipid droplet accumulation in the liver.

### 2.6 Biochemical analysis

Fasting blood glucose (FBG) levels were detected using a Life Scan One Touch Ultra Easy glucometer (Wayne, PA, USA), and serum insulin levels were detected using an insulin mouse ELISA kit according to the manufacturer’s instructions. And the homeostastic model assessment-insulin resistance (HOMA-IR) index was calculated according to the following formula: FBG × fasting serum insulin/22.5 [41]. Blood-biochemical indicators, including serum TG, TC, alanine transaminase (ALT) and aspartate transaminase (AST), were measured using a Beckman automatic biochemistry analyzer (Palo Alto, CA, USA). The levels of TG, TC and NEFA in the liver or primary hepatocytes were detected using commercial kits according to the manufacturer’s instructions. Hepatic hydroxyproline levels were determined to evaluate hepatic fibrosis using a commercial kit. Briefly, fresh liver homogenates were hydrolyzed in 12 mol/L hydrochloric acid at 120°C for 3 h, incubated with chloramine T and 4-(dimethylamino) benzaldehyde and then subjected to absorbance detection at 560 nm. The levels of inflammatory cytokines in the liver or cell culture were measured using commercial ELISA kits according to the manufacturer’s instructions. LDH releases in the medium and hepatic irisin levels were measured using commercial kits following the manufacturer’s instructions.

### 2.7 Primary hepatocytes isolation and treatments

Primary hepatocytes were isolated as previously described [29]. Briefly, the livers were harvested from 8-week- old C57BL/6 mice and perfused with collagenase buffer to isolate the hepatocytes. Next, the cell suspensions were centrifuged at 50 g for 2 min and purified with a 50% Percoll solution. To imitate NAFLD in vitro, primary hepatocytes were stimulated with 0.5 mmol/L palmitic acid plus 1.0 mmol/L oleic acid (PO) for 24 h, while cells in the control group were incubated with an equal amount of fatty acid free bovine serum albumin (BSA) [29]. To manipulate miR-665-3p levels in hepatocytes, cells were transfected with the miR-665-3p agomir, miR-665-3p antagomir or matched negative controls at a dose of 50 nmol/L using a Lipofectamine 6000^TM^ reagent for 24 h before PO stimulation. To inhibit AMPKα, cells were treated with CpC (10 μmol/L) for 24 h along with PO or BSA stimulation [42].

### 2.8 Analysis of oxidative stress

Intracellular ROS level was measured using a DCFH-DA probe according to previous studies [43–45]. Briefly, the livers or hepatocytes were lysed, incubated with 50 μmol/L DCFH-DA at 37°C for 1 h, and then the absorbance was measured at the excitation/emission wavelength of 485/535 nm. Hepatic H_2_O_2_ and MDA levels were detected using commercial kits according to the manufacturer’s instructions.

### 2.9 Luciferase reporter assay

Vectors containing the wild type (WT) or truncated (TRU) FNDC5 3ʹ-UTR were purchased from Shanghai GenePharma Co.,Ltd., and cotransfected with the miR-665-3p agomir (50 nmol/L) into HEK293T cells for 48 h. Then, the cells were lysed, with Firefly and Renilla luciferase activities measured using a Dual-Luciferase Reporter Assay System (Promega). Data were expressed as the Renilla luciferase activity-to-Firefly luciferase activity ratio [46–48].

### 2.10 Statistical analysis

All results were expressed as the means ± standard deviations, and statistical analyses were performed using a SPSS software (Version 19.0). Differences between 2 groups were compared using an unpaired two-tailed Student’s *t*-test, while differences among 3 or more groups were compared using the one-way analysis of variance followed by Tukey post-hoc test. *P*-values less than 0.05 were considered statistically significant.

## 3. Results

In the present study, we aim to investigate the role of miR-665-3p in oxidative stress, inflammation and NAFLD progression by in vivo and in vitro studies, and elucidate whether these effects are mediated by AMPKα. A significant upregulation of miR-665-3p level was measured in the liver and primary hepatocytes upon HFD or PO stimulation, and the elevated miR-665-3p expression aggravated oxidative stress, inflammation and NAFLD progression in mice. In contrast, miR-665-3p inhibition by the miR-665-3p antagomir significantly prevented HFD-induced oxidative stress, inflammation and hepatic dysfunction in vivo. Manipulation of miR-665-3p in primary hepatocytes also caused similar phenotypic alterations in vitro. Mechanistically, we demonstrated that miR-665-3p directly bound to the 3ʹ-UTR of FNDC5 to downregulate its expression and inactivated the downstream AMPKα pathway, thereby facilitating oxidative stress, inflammation and NAFLD progression.

### 3.1 miR-665-3p antagomir alleviates NAFLD progression in mice

**Figure 1.**
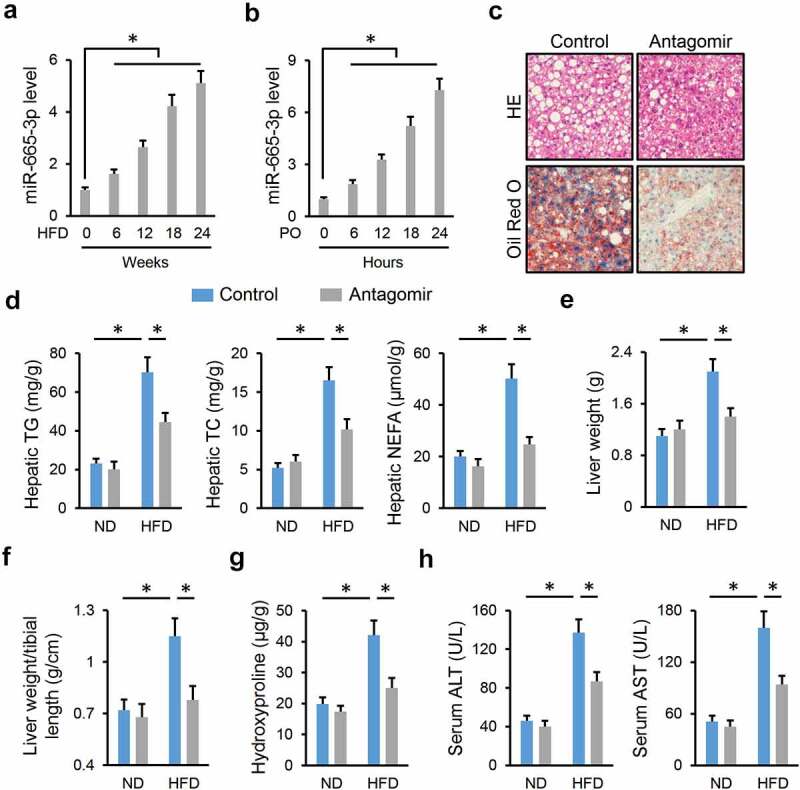
**miR-665-3p antagomir alleviates NAFLD progression in mice**. (a) The levels of miR-665-3p in HFD mice. (b) The levels of miR-665-3p PO-stimulated primary hepatocytes. (c) HE and oil red O staining of liver tissues. (d) Hepatic levels of TG, TC and NEFA. (e-f) Quantification of the liver weight and liver weight/tibial length. (g) Hepatic hydroxyproline levels. (h) Serum ALT and AST levels. All results were expressed as the means ± standard deviations, n = 6 for each group, and *P < 0.05 was considered statistically significant. n.s. indicated no significance.

To determine the involvement of miR-665-3p in NAFLD progression, we first measured whether miR-665-3p expression was altered during NAFLD progression. As shown in Figure 1(a), hepatic miR-665-3p level was significantly increased in HFD-treated mice. In addition, the level of miR-665-3p was also elevated in PO-stimulated primary hepatocytes (Figure 1(b)). The dramatic increase of miR-665-3p level during NAFLD progression promoted us to explore whether inhibiting miR-665-3p could alleviate HFD-induced NAFLD. As shown in Figure S1A, hepatic miR-665-3p expression was inhibited by the miR-665-3p antagomir. Intriguingly, treatment with the miR-665-3p antagomir significantly reduced body weight in HFD mice, and the weights of epididymal and inguinal fat pads were also decreased (Figure S1B-C). However, food intake in mice with either ND or HFD stimulation was unaffected by the miR-665-3p antagomir (Figure S1D). As shown in Figure S1E-G, the miR-665-3p antagomir dramatically restored HFD-induced glycometabolic disorder, as evidenced by the decreased FBG, serum insulin and HOMA-IR levels. Meanwhile, hyperlipemia in HFD mice was also improved in those treated with the miR-665-3p antagomir (Figure S1H). Consistent with the alterations of systemic metabolism, the miR-665-3p antagomir also decreased lipid deposition in the liver upon HFD stimulation, as evidenced by HE/Oil red O staining and the decreased hepatic TG, TC and NEFA contents (Figure 1(c-d)). Accordingly, HFD-induced increases in liver weight and liver weight/tibial length were prevented by the miR-665-3p antagomir (Figure 1(e-f)). Fibrosis is a critical pathology of NAFLD, and our findings indicated that the miR-665-3p antagomir dramatically ameliorated HFD-induced hepatic fibrosis, as evidenced by the decreased hepatic hydroxyproline content and mRNA levels of fibrotic markers, including Col1α1, Col3α1, CTGF and TGF-β1 (Figure 1(g) and Figure S1I). Consistently, serum levels of ALT and AST, two biomarkers of liver injury, were significantly lower in the miR-665-3p antagomir-treated mice upon HFD stimulation (Figure 1(h)). These findings indicate that the miR-665-3p antagomir alleviates NAFLD progression in mice.

### 3.2 miR-665-3p antagomir reduces HFD-induced hepatic oxidative stress and inflammation

**Figure 2.**
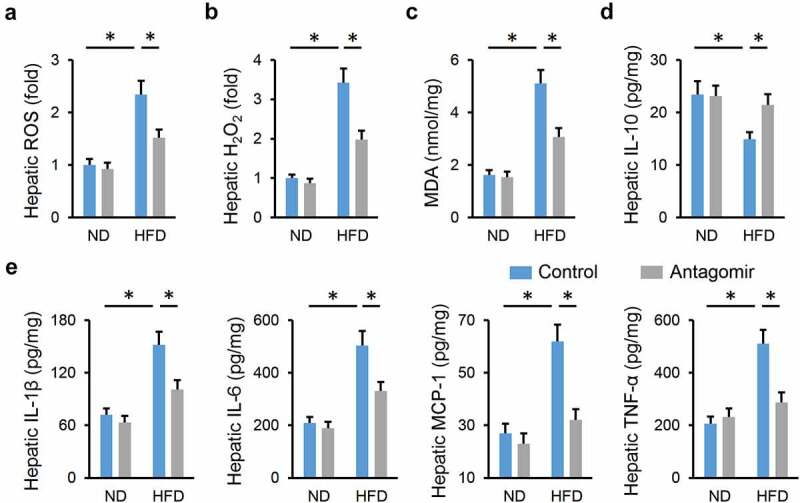
**miR-665-3p antagomir reduces HFD-induced hepatic oxidative stress and inflammation**. (a) Hepatic ROS levels determined by DCFH-DA probe. (b) H_2_O_2_ levels in the liver. (c) MDA levels in the liver. (d-e) Hepatic IL-1β, IL-6, MCP-1, TNF-α and IL-10 levels determined by the commercial ELISA kits. All results were expressed as the means ± standard deviations, n = 6 for each group, and *P < 0.05 was considered statistically significant.

Emerging studies have identified oxidative stress and chronic inflammation as major culprits of NAFLD; therefore, we detected whether treatment with the miR-665-3p antagomir could reduce HFD-induced hepatic oxidative stress and inflammation. As shown in Figure 2(a-b), hepatic ROS and H_2_O_2_ levels were significantly decreased by the miR-665-3p antagomir upon HFD stimulation. And the miR-665-3p antagomir also reduced hepatic levels of MDA, a product of lipid peroxidation and a biomarker for oxidative stress (Figure 2(c)). In addition, the level of antiinflammatory cytokine (IL-10) was increased, whereas the levels of proinflammatory cytokines (IL-1β, IL-6, MCP-1 and TNF-α) were decreased in the miR-665-3p antagomir-treated livers upon HFD stimulation (Figure 2(d-e)). Collectively, our results reveal that the miR-665-3p antagomir reduces HFD-induced hepatic oxidative stress and inflammation.

### 3.3 miR-665-3p agomir facilitates NAFLD progression in mice

**Figure 3.**
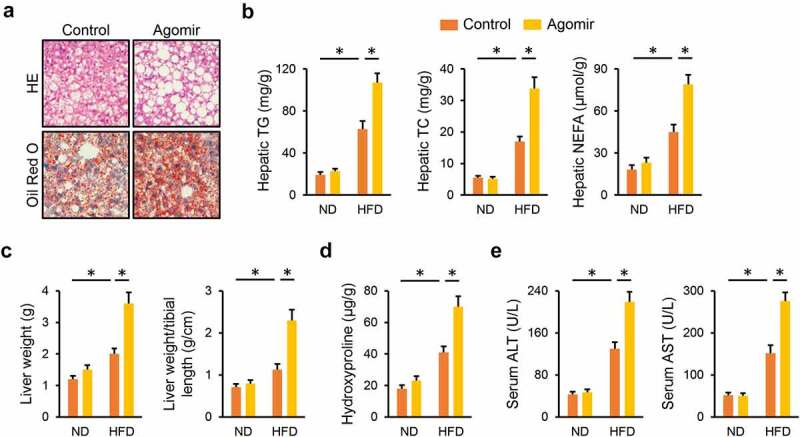
**miR-665-3p agomir facilitates NAFLD progression in mice**. (a) HE and oil red O staining of liver tissues. (b) Hepatic levels of TG, TC and NEFA. (c) Quantification of the liver weight and liver weight/tibial length. (d) Hepatic hydroxyproline levels. (e) Serum ALT and AST levels. All results were expressed as the means ± standard deviations, n = 6 for each group, and *P < 0.05 was considered statistically significant.

Next, we determined whether elevating miR-665-3p levels contributed to the progression of NAFLD. As shown in Figure S2A, hepatic miR-665-3p level was significantly increased by the miR-665-3p agomir. HFD-induced increases of body weight and adipose tissue weight were further enhanced in mice treated with the miR-665-3p agomir (Figure S2B-C). And systemic metabolic disorders of glucose and lipid were also amplified by the miR-665-3p agomir upon HFD stimulation, as evidenced by the increased levels of FBG, serum insulin, HOMA-IR, serum TG and TC (Figure S2D-G). In addition, treatment with the miR-665-3p agomir further aggravated lipid accumulation in the liver from HFD mice, and increased liver weight (Figure 3(a-c)). As expected, hepatic fibrosis and injury were exacerbated in the miR-665-3p agomir-treated HFD mice, as evidenced by the increased levels of hepatic hydroxyproline and serum ALT and AST (Figure 3(d-e)). Taken together, our findings suggest that the miR-665-3p agomir facilitates NAFLD progression in mice.

### 3.4 miR-665-3p agomir aggravates HFD-induced hepatic oxidative stress and inflammation

**Figure 4.**
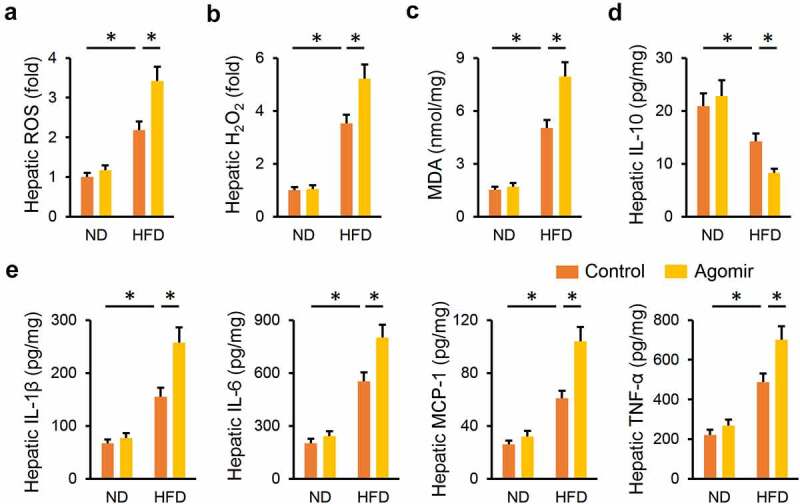
**miR-665-3p agomir aggravates HFD-induced hepatic oxidative stress and inflammation**. (a) Hepatic ROS levels determined by DCFH-DA probe. (b) H_2_O_2_ levels in the liver. (c) MDA levels in the liver. (d-e) Hepatic IL-1β, IL-6, MCP-1, TNF-α and IL-10 levels determined by the commercial ELISA kits. All results were expressed as the means ± standard deviations, n = 6 for each group, and *P < 0.05 was considered statistically significant.

Consistent with the phenotypic alterations, the miR-665-3p agomir significantly increased HFD-induced generations of hepatic ROS and H_2_O_2_ (Figure 4(a-b)). Meanwhile, MDA production in the liver was also enhanced by the miR-665-3p agomir upon HFD stimulation (Figure 4(c)). In addition, the miR-665-3p agomir also facilitated HFD-induced hepatic inflammation, as evidenced by the decreased hepatic IL-18 level, and increased hepatic IL-1β, IL-6, MCP-1 and TNF-α levels (Figure 4(d-e)). These data reveal that the miR-665-3p agomir aggravates HFD-induced hepatic oxidative stress and inflammation.

### 3.5 miR-665-3p modulates oxidative stress, inflammation and cellular injury in PO-treated primary hepatocytes

**Figure 5.**
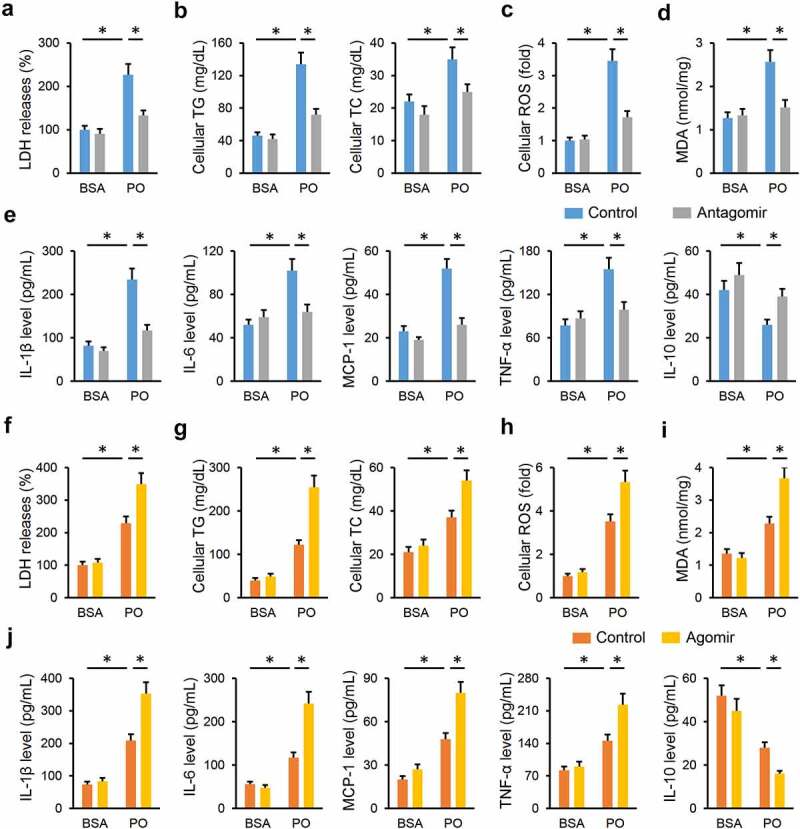
**miR-665-3p modulates oxidative stress, inflammation and cellular injury in PO-treated primary hepatocytes**. (a) LDH releases in PO-stimulated hepatocytes treated with or without the miR-665-3p antagomir. (b) Intracellular TG and TC levels in the miR-665-3p antagomir-treated hepatocytes upon PO stimulation. (c) Cellular ROS levels determined by DCFH-DA probe. (d) MDA levels in the miR-665-3p antagomir-treated hepatocytes. (e) The levels of IL-1β, IL-6, MCP-1, TNF-α and IL-10 in the miR-665-3p antagomir-treated hepatocytes. (f) LDH releases in PO-stimulated hepatocytes treated with or without the miR-665-3p agomir. (g) Intracellular TG and TC levels in the miR-665-3p agomir-treated hepatocytes upon PO stimulation. (h) Cellular ROS levels determined by DCFH-DA probe. (i) MDA levels in the miR-665-3p agomir-treated hepatocytes. (j) The levels of IL-1β, IL-6, MCP-1, TNF-α and IL-10 in the miR-665-3p agomir-treated hepatocytes. All results were expressed as the means ± standard deviations, n = 6 for each group, and *P < 0.05 was considered statistically significant.

Next, we tried to validate the role of miR-665-3p in primary hepatocytes in vitro. As shown in Figure 5(a), the miR-665-3p antagomir significantly reduced LDH releases in PO-treated hepatocytes. And intracellular accumulations of TG and TC were also decreased in the miR-665-3p antagomir-treated hepatocytes upon PO stimulation (Figure 5(b)). As expected, treatment with the miR-665-3p antagomir dramatically suppressed PO-stimulated oxidative stress in primary hepatocytes, as evidenced by the decreased cellular ROS and MDA levels (Figure 5(c-d)). And PO-induced increases of IL-1β, IL-6, MCP-1 and TNF-α, and decrease of IL-10 were significantly attenuated by the miR-665-3p antagomir (Figure 5e). In contrast, PO-induced elevation of LDH releases in primary hepatocytes was further increased by the miR-665-3p agomir (figure 5(f)). And treatment with the miR-665-3p agomir also facilitated intracellular TG and TC accumulations (Figure 5(g)). Consistent with the phenotypic alterations, the miR-665-3p agomir also aggravated oxidative damage and inflammatory response in PO-treated primary hepatocytes (Figure 5(h-j)). These results implicate that miR-665-3p modulates oxidative stress, inflammation and cellular injury in PO-treated primary hepatocytes.

### 3.6 miR-665-3p antagomir prevents NAFLD progression via activating AMPKα in vivo and in vitro

**Figure 6.**
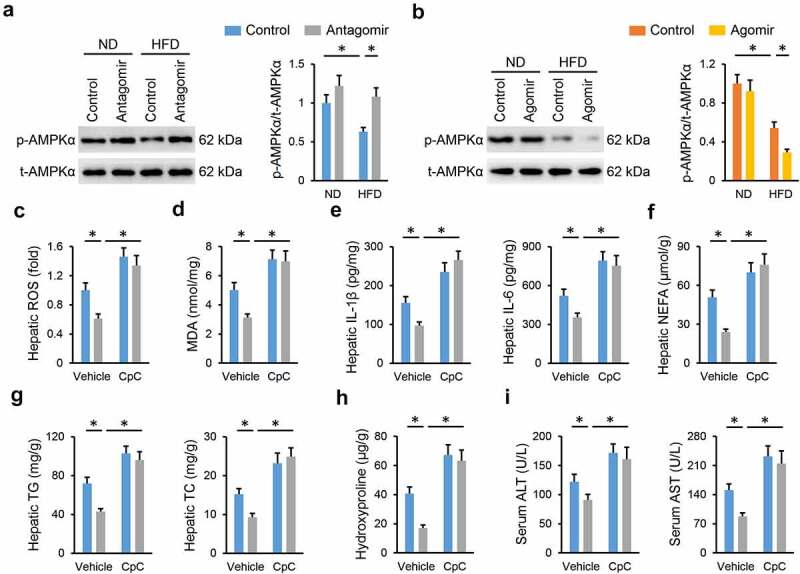
**miR-665-3p antagomir prevents NAFLD progression via activating AMPKα in vivo**. (a-b) The levels of AMPKα phosphorylation. (c) Hepatic ROS levels determined by DCFH-DA probe. (d) MDA levels in the liver. (e) Hepatic IL-1β and IL-6 levels determined by the commercial ELISA kits. (f-g) Hepatic levels of TG, TC and NEFA. (h) Hepatic hydroxyproline levels. (i) Serum ALT and AST levels. All results were expressed as the means ± standard deviations, n = 6 for each group, and *P < 0.05 was considered statistically significant.

**Figure 7.**
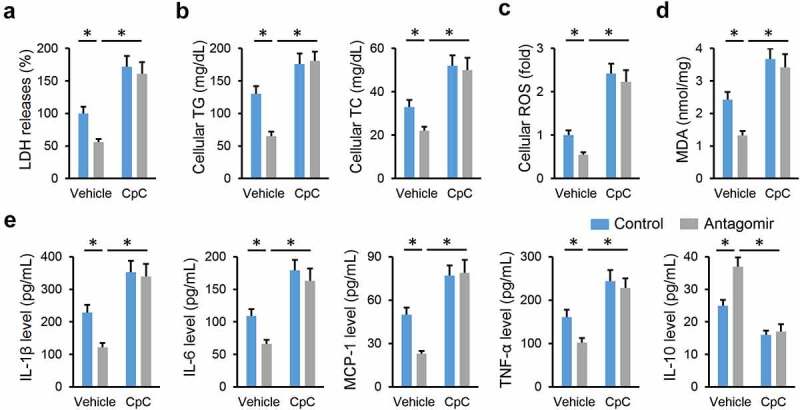
**miR-665-3p antagomir prevents NAFLD progression via activating AMPKα in vitro**. (a) LDH releases in the miR-665-3p antagomir-treated hepatocytes with or without CpC incubated upon PO stimulation. (b) Intracellular TG and TC levels. (c) Cellular ROS levels determined by DCFH-DA probe. (d) MDA levels. (e) The levels of IL-1β, IL-6, MCP-1, TNF-α and IL-10 in the miR-665-3p antagomir-treated hepatocytes with or without CpC incubated upon PO stimulation. All results were expressed as the means ± standard deviations, n = 6 for each group, and *P < 0.05 was considered statistically significant.

AMPKα is a promising therapeutic target to treat NAFLD; therefore, we investigated whether the miR-665-3p antagomir prevented NAFLD progression via activating AMPKα. As shown in Figure 6(a-b), the miR-665-3p antagomir restored, while the miR-665-3p agomir further decreased AMPKα phosphorylation in the liver from HFD mice. To validate the necessity of AMPKα, HFD mice were pretreated with CpC to inhibit AMPKα. As shown in Figure 6(c-e), the miR-665-3p antagomir-induced inhibition on oxidative stress and inflammation in HFD mice were completely abolished by CpC treatment. The miR-665-3p antagomir significantly reduced hepatic lipid accumulation in HFD mice, but had no effect in those treated with CpC (figure 6(f-g)). Accordingly, AMPKα inhibition also abrogated the protective effects of the miR-665-3p antagomir against HFD-induced hepatic fibrosis and injury, as evidenced by the increased hepatic hydroxyproline content and serum ALT and AST levels (Figure 6(h-i)). In line with the in vivo results, the inhibitory effects of the miR-665-3p antagomir on PO-induced LDH releases and intracellular lipid accumulations were completely abolished by CpC (Figure 7(a-b)). In addition, CpC treatment also blocked the antioxidant and antiinflammatory effects of the miR-665-3p antagomir in PO-stimulated primary hepatocytes (Figure 7(c-e)). Together, these findings suggest that the miR-665-3p antagomir prevents NAFLD progression via activating AMPKα in vivo and in vitro.

### 3.7 miR-665-3p antagomir activates AMPKα via increasing FNDC5 expression

**Figure 8.**
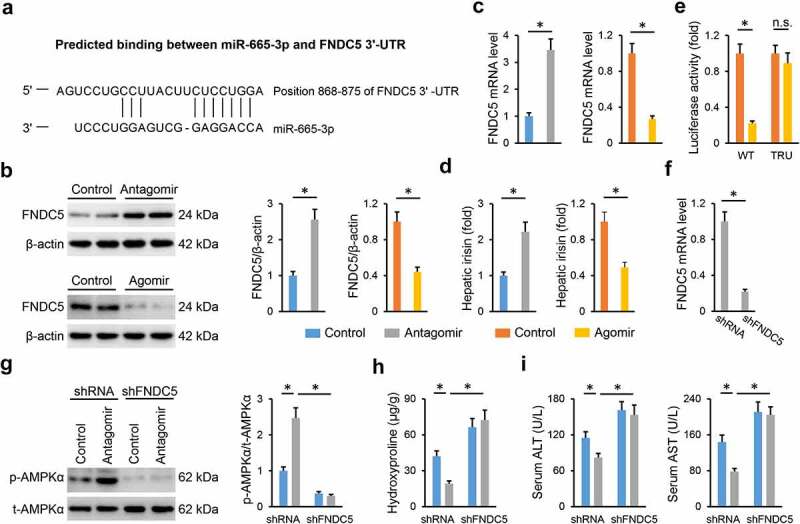
**miR-665-3p antagomir activates AMPKα via increasing FNDC5 expression**. (a) Graphic representation of the miR-665-3p binding motifs within the 3ʹ-UTR of FNDC5. (b-c) The mRNA and protein levels of FNDC5 in the liver from HFD mice. (d) The levels of hepatic irisin detected by a commercial ELISA kit. (e) Relative luciferase activity of the reporter constructs containing either WT or TRU 3ʹ-UTR of FNDC5 after treatment with miR-665-3p agomir. (f) The mRNA levels of FNDC5 in the liver from HFD mice treated with shFNDC5 or shRNA. (g) The levels of AMPKα phosphorylation. (h) Hepatic hydroxyproline levels. (i) Serum ALT and AST levels. All results were expressed as the means ± standard deviations, n = 6 for each group, and *P < 0.05 was considered statistically significant.

Finally, we explored the underlying mechanism through which the miR-665-3p antagomir activated AMPKα. As predicted by the TargetScan online software, FNDC5 was identified as a potential candidate for its role in regulating NAFLD progression and AMPKα activation [49,50]. A predicted binding site of miR-665-3p was found in FNDC5 3ʹ-UTR (Figure 8(a)). In addition, we observed that the miR-665-3p antagomir increased, whereas the miR-665-3p agomir decreased hepatic FNDC5 mRNA and protein levels in HFD mice (Figure 8(b-c)). Meanwhile, hepatic levels of irisin, the cleaved and active form of FNDC5, were also increased by the miR-665-3p antagomir, but decreased by the miR-665-3p agomir (Figure 8(d)). Next, luciferase reporter assay was performed to determine the direct interaction between miR-665-3p and FNDC5. As shown in Figure 8(e), the miR-665-3p agomir significantly reduced the luciferase activities in cells transfected with WT FNDC5 3ʹ-UTR, but not in those transfected with TRU FNDC5 3ʹ-UTR. To validate the involvement of FNDC5, mice were intravenously injected with shFNDC5 to knock down endogenous FNDC5 expression in the liver (Figure 8(f)). As shown in Figure 8(g), FNDC5 silence blocked AMPKα activation in the miR-665-3p antagomir-treated HFD mice. Accordingly, the miR-665-3p antagomir-mediated hepatoprotection was also abrogated by FNDC5 knockdown, as evidenced by the increased hepatic hydroxyproline content and serum ALT and AST levels (Figure 8(h-i)). In conclusion, our results show that the miR-665-3p antagomir activates AMPKα via increasing FNDC5 expression.

## 4. Discussion

**Figure 9.**
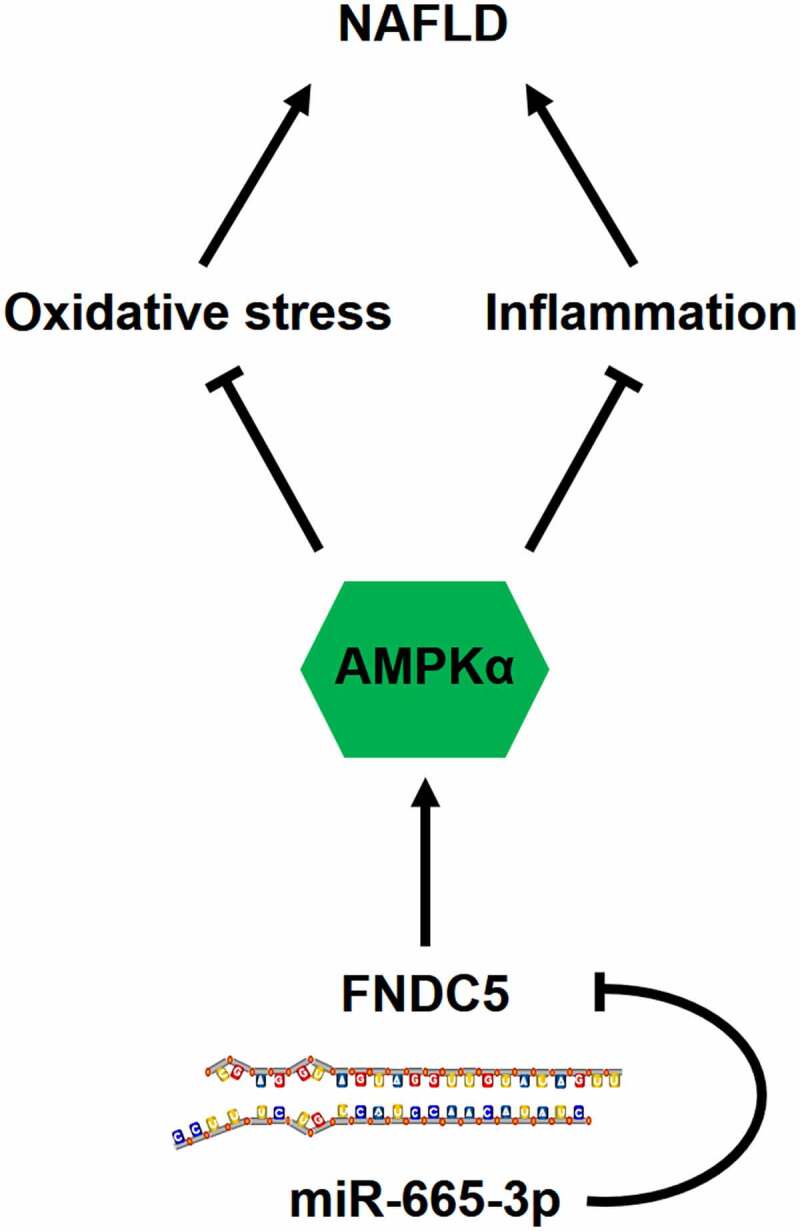
**A diagram of the mechanisms of miR-665-3p in the pathogenesis of NAFLD**. miR-665-3p directly binds to the 3ʹ-UTR of FNDC5 and inhibit its expression, thereby exacerbating oxidative stress and inflammation via inactivating AMPKα pathway during NAFLD.

Due to the dramatic changes in diets and lifestyles, NAFLD has becoming one of the leading chronic liver diseases, affecting about 1.7 billion individuals worldwide, and the national prevalence of NAFLD in China is approximately 29.2% [1,51,52]. The spectrum of this disease ranges from simple hepatic steatosis to advanced nonalcoholic steatohepatitis that eventually progresses to end-stage liver diseases such as cirrhosis and hepatocellular carcinoma. Moreover, a large proportion of NAFLD patients also suffer from a high prevalence of extrahepatic complications (e.g., cardiovascular diseases and extrahepatic cancers). Yet, there are no effective strategies to manage NAFLD and its related comorbidities. In the present study, we measure a significant upregulation of miR-665-3p level in the liver and primary hepatocytes upon HFD or PO stimulation, and the elevated miR-665-3p expression aggravates oxidative stress, inflammation and NAFLD progression in mice. In contrast, miR-665-3p inhibition by the miR-665-3p antagomir significantly prevents HFD-induced oxidative stress, inflammation and hepatic dysfunction in vivo. Manipulation of miR-665-3p in primary hepatocytes also causes similar phenotypic alterations in vitro. Mechanistically, we demonstrate that miR-665-3p directly binds to the 3ʹ-UTR of FNDC5 to downregulate its expression and inactivates the downstream AMPKα pathway, thereby facilitating oxidative stress, inflammation and NAFLD progression (Figure 9). Overall, our findings identify miR-665-3p as an endogenous positive regulator of NAFLD via inactivating FNDC5/AMPKα pathway, and inhibiting miR-665-3p may provide novel therapeutic strategies to treat NAFLD.

The liver is a key organ to control systemic glycolipid metabolism and the whole-body energy homeostasis, while its function is evidently compromised in the development of NAFLD due to the excessive lipid deposition and hepatic fibrosis. Accumulation of NEFA in hepatocytes interferes the electron transport chain and mitochondrial function, causing electron leakage and uncontrolled ROS generation. In addition, NEFA and intracellular substances derived from the injured hepatocytes activate intrahepatic Kupffer cells to aggravate hepatic inflammation [17,53,54]. Unfortunately, oxidative stress and inflammation form a vicious cycle to further accelerate NAFLD progression. Moreover, previous findings by us (unpublished) and the others have determined a compromised antioxidant capacity in the liver from HFD mice [7,54]. Hepatic fibrosis is a key feature of NAFLD, and closely correlates with the liver function and prognosis in NAFLD patients. During NAFLD progression, hepatic stellate cells are activated and transform into proliferative, fibrogenic and contractile myofibroblasts, eventually resulting in excessive synthesis and deposition of extracellular matrix [55,56]. Herein, we demonstrated that the miR-665-3p antagomir significantly inhibited oxidative stress and inflammation, thereby attenuating HFD-induced hepatic fibrosis and dysfunction. The liver also plays critical roles in maintaining systemic glycolipid metabolism. In line with the improved NAFLD and hepatic function, HFD-induced glycometabolic disorder (e.g., adipose tissue weight, FBG, serum insulin, and HOMA-IR levels) was also alleviated by systemic injections of the miR-665-3p antagomir, yet exacerbated by the miR-665-3p agomir. These data suggest that inhibiting miR-665-3p may provide beneficial effects on both hepatic function and systemic glycometabolic disorder in NAFLD patients.

FNDC5 is a widely distributed type I transmembrane glycoprotein, and can be proteolytically cleaved and released as irisin, an exercise-responsive polypeptide myokine [57,58]. Emerging studies have revealed a fact that FNDC5 and its cleaved form, irisin, play critical roles in metabolic diseases, including NAFLD [49,59]. Zhang et al previously reported that serum irisin levels were decreased in NAFLD patients, and negatively correlated with intrahepatic TG contents [60]. And Canivet et al demonstrated that the liver and hepatocytes are critical sources of FNDC5 and irisin, and that local FNDC5 in the liver stimulated significant benefits against hepatic steatosis and hepatocyte apoptosis [49]. In addition, FNDC5 and irisin are also implicated in the regulation of oxidative stress and inflammation in different organs, including the liver. Zhang et al recently found that FNDC5 suppressed protein kinase B-dependent nuclear export and degradation of nuclear factor erythroid-2-related factor-2, thereby alleviating doxorubicin-induced oxidative stress in the heart [61]. Bi et al showed that treatment with exogenous irisin protected against I/R-induced mitochondrial injury and oxidative stress in the liver [62]. Moreover, serum irisin levels were found to be independently associated with portal inflammation and oxidative status in NAFLD progression [63,64]. A very recent study by Li et al proved that FNDC5 upregulation by nicotinamide riboside significantly prevented HFD-induced NAFLD [65]. AMPKα is identified as a critical downstream target of FNDC5 and irisin, and mediates their antioxidant and antiinflammatory effects upon different stimulations [50,66]. Herein, we showed that the miR-655-3p antagomir activated AMPKα via upregulating FNDC5, and FNDC5 silence blocked the miR-655-3p antagomir-mediated AMPKα activation and hepatoprotective effects against NAFLD.

## 5. Conclusion

In summary, we for the first time demonstrate the involvement of miR-665-3p in promoting NAFLD via inactivating FNDC5/AMPKα pathway, and inhibiting miR-665-3p may provide novel therapeutic strategies to treat NAFLD.

## Supplementary Material

Supplemental Material

## Data Availability

The data that support the findings of this study are available from the corresponding author upon reasonable request.

## Highlights

1. miR-665-3p level is upregulated during NAFLD progression.
2. miR-665-3p elevation aggravates oxidative stress, inflammation and NAFLD.
3. miR-665-3p inhibition prevents oxidative stress, inflammation and NAFLD.
4. miR-665-3p binds to FNDC5 3ʹ-UTR and subsequently inactivates AMPKα pathway.

## References

[1] Zhou J, Zhou F, Wang W, et al. Epidemiological features of NAFLD from 1999 to 2018 in China. Hepatology. 2020;71:1851–1864. 32012320 10.1002/hep.31150

[2] Wu Z, Ma H, Wang L, et al. Tumor suppressor ZHX2 inhibits NAFLD-HCC progression via blocking LPL-mediated lipid uptake. Cell Death Differ. 2020;27:1693–1708. 31740790 10.1038/s41418-019-0453-z PMC7206072

[3] Qian LL, Wu L, Zhang L, et al. Serum biomarkers combined with ultrasonography for early diagnosis of non-alcoholic fatty liver disease confirmed by magnetic resonance spectroscopy. Acta Pharmacol Sin. 2020;41:554–560. 31776449 10.1038/s41401-019-0321-x PMC7471465

[4] Schulien I, Hockenjos B, Schmitt-Graeff A, et al. The transcription factor c-Jun/AP-1 promotes liver fibrosis during non-alcoholic steatohepatitis by regulating Osteopontin expression. Cell Death Differ. 2019;26:1688–1699. 30778201 10.1038/s41418-018-0239-8 PMC6748141

[5] Chen X, Xue H, Fang W, et al. Adropin protects against liver injury in nonalcoholic steatohepatitis via the Nrf2 mediated antioxidant capacity. Redox Biol. 2019;21:101068. 30684890 10.1016/j.redox.2018.101068 PMC6351233

[6] Brandt A, Nier A, Jin CJ, et al. Consumption of decaffeinated coffee protects against the development of early non-alcoholic steatohepatitis: role of intestinal barrier function. Redox Biol. 2019;21:101092. 30605883 10.1016/j.redox.2018.101092 PMC6313826

[7] Kim MH, Seong JB, Huh JW, et al. Peroxiredoxin 5 ameliorates obesity-induced non-alcoholic fatty liver disease through the regulation of oxidative stress and AMP-activated protein kinase signaling. Redox Biol. 2020;28:101315. 31505325 10.1016/j.redox.2019.101315 PMC6736789

[8] Tan M, Mosaoa R, Graham GT, et al. Inhibition of the mitochondrial citrate carrier, Slc25a1, reverts steatosis, glucose intolerance, and inflammation in preclinical models of NAFLD/NASH. Cell Death Differ. 2020;27:2143–2157. 31959914 10.1038/s41418-020-0491-6 PMC7308387

[9] Saito Y, Hikita H, Nozaki Y, et al. DNase II activated by the mitochondrial apoptotic pathway regulates RIP1-dependent non-apoptotic hepatocyte death via the TLR9/IFN-beta signaling pathway. Cell Death Differ. 2019;26:470–486. 29855540 10.1038/s41418-018-0131-6 PMC6370801

[10] Xu DQ, Li CJ, Jiang ZZ, et al. The hypoglycemic mechanism of catalpol involves increased AMPK-mediated mitochondrial biogenesis. Acta Pharmacol Sin. 2020;41:791–799. 31937931 10.1038/s41401-019-0345-2 PMC7470840

[11] Zhang X, Ma ZG, Yuan YP, et al. Rosmarinic acid attenuates cardiac fibrosis following long-term pressure overload via AMPKalpha/Smad3 signaling. Cell Death Dis. 2018;9:102. 29367637 10.1038/s41419-017-0123-3 PMC5833382

[12] Zhang T, Liu J, Shen S, et al. SIRT3 promotes lipophagy and chaperon-mediated autophagy to protect hepatocytes against lipotoxicity. Cell Death Differ. 2020;27:329–344. 31160717 10.1038/s41418-019-0356-z PMC7206074

[13] Gao J, Yuan J, Wang Q, et al. Metformin protects against PM2.5-induced lung injury and cardiac dysfunction independent of AMP-activated protein kinase alpha2. Redox Biol. 2020;28:101345. 31669973 10.1016/j.redox.2019.101345 PMC6838896

[14] Yang L, Li X, Jiang A, et al. Metformin alleviates lead-induced mitochondrial fragmentation via AMPK/Nrf2 activation in SH-SY5Y cells. Redox Biol. 2020;36:101626. 32863218 10.1016/j.redox.2020.101626 PMC7334619

[15] Hu C, Zhang X, Wei W, et al. Matrine attenuates oxidative stress and cardiomyocyte apoptosis in doxorubicin-induced cardiotoxicity via maintaining AMPKalpha/UCP2 pathway. Acta Pharm Sin B. 2019;9:690–701. 31384530 10.1016/j.apsb.2019.03.003 PMC6664099

[16] Han CJ, Zheng JY, Sun L, et al. The oncometabolite 2-hydroxyglutarate inhibits microglial activation via the AMPK/mTOR/NF-kappaB pathway. Acta Pharmacol Sin. 2019;40:1292–1302. 31015738 10.1038/s41401-019-0225-9 PMC6786375

[17] Garcia D, Hellberg K, Chaix A, et al. Genetic liver-specific AMPK activation protects against diet-induced obesity and NAFLD. Cell Rep. 2019;26:192–208. 30605676 10.1016/j.celrep.2018.12.036 PMC6344045

[18] Zhu Y, Gu L, Lin X, et al. LINC00265 promotes colorectal tumorigenesis via ZMIZ2 and USP7-mediated stabilization of beta-catenin. Cell Death Differ. 2020;27:1316–1327. 31527801 10.1038/s41418-019-0417-3 PMC7206056

[19] Liu HM, Jia Y, Zhang YX, et al. Dysregulation of miR-135a-5p promotes the development of rat pulmonary arterial hypertension in vivo and in vitro. Acta Pharmacol Sin. 2019;40:477–485. 30038339 10.1038/s41401-018-0076-9 PMC6462033

[20] Ren X, Chen Z, Ruan J, et al. Trichloroethylene-induced downregulation of miR-199b-5p contributes to SET-mediated apoptosis in hepatocytes. Cell Biol Toxicol. 2019;35:565–572. 31140026 10.1007/s10565-019-09479-3

[21] Rahbarghazi R, Keyhanmanesh R, Rezaie J, et al. c-kit+ cells offer hopes in ameliorating asthmatic pathologies via regulation of miRNA-133 and miRNA-126. Iran J Basic Med Sci. 2021;24:369–376. 33995948 10.22038/ijbms.2021.49008.11231 PMC8087855

[22] Alamdari AF, Rahnemayan S, Rajabi H, et al. Melatonin as a promising modulator of aging related neurodegenerative disorders: role of microRNAs. Pharmacol Res. 2021;173:105839. 34418564 10.1016/j.phrs.2021.105839

[23] Gjorgjieva M, Sobolewski C, Dolicka D, et al. miRNAs and NAFLD: from pathophysiology to therapy. Gut. 2019;68:2065–2079. 31300518 10.1136/gutjnl-2018-318146

[24] Li Z, Wang G, Feng D, et al. Targeting the miR-665-3p-ATG4B-autophagy axis relieves inflammation and apoptosis in intestinal ischemia/reperfusion. Cell Death Dis. 2018;9:483. 29706629 10.1038/s41419-018-0518-9 PMC5924757

[25] Guo Q, Lin Y, Hu J. Inhibition of miR-665-3p enhances autophagy and alleviates inflammation in fusarium solani-induced keratitis. Invest Ophthalmol Vis Sci. 2021;62:24. 10.1167/iovs.62.1.24 PMC7838549 33481985

[26] Zhang X, Feng Y, Li J, et al. MicroRNA-665-3p attenuates oxygen-glucose deprivation-evoked microglial cell apoptosis and inflammatory response by inhibiting NF-kappaB signaling via targeting TRIM8. Int Immunopharmacol. 2020;85:106650. 32512270 10.1016/j.intimp.2020.106650

[27] Lin B, Feng DG, Xu J. microRNA-665 silencing improves cardiac function in rats with heart failure through activation of the cAMP signaling pathway. J Cell Physiol. 2019;234:13169–13181. 30666648 10.1002/jcp.27987 PMC13483206

[28] Yu J, Yang W, Wang W, et al. Involvement of miR-665 in protection effect of dexmedetomidine against oxidative stress injury in myocardial cells via CB2 and CK1. Biomed Pharmacother. 2019;115:108894. 31026731 10.1016/j.biopha.2019.108894

[29] Tong J, Han CJ, Zhang JZ, et al. Hepatic interferon regulatory factor 6 alleviates liver steatosis and metabolic disorder by transcriptionally suppressing Peroxisome proliferator-activated receptor gamma in mice. Hepatology. 2019;69:2471–2488. 30748020 10.1002/hep.30559

[30] Ma ZG, Dai J, Zhang WB, et al. Protection against cardiac hypertrophy by geniposide involves the GLP-1 receptor/AMPKalpha signalling pathway. Br J Pharmacol. 2016;173:1502–1516. 26845648 10.1111/bph.13449 PMC4831312

[31] Hu C, Zhang X, Song P, et al. Meteorin-like protein attenuates doxorubicin-induced cardiotoxicity via activating cAMP/PKA/SIRT1 pathway. Redox Biol. 2020;37:101747. 33045622 10.1016/j.redox.2020.101747 PMC7558217

[32] Zhang X, Hu C, Yuan XP, et al. Osteocrin, a novel myokine, prevents diabetic cardiomyopathy via restoring proteasomal activity. Cell Death Dis. 2021;12:624. 34135313 10.1038/s41419-021-03922-2 PMC8209005

[33] Liu FY, Fan D, Yang Z, et al. TLR9 is essential for HMGB1-mediated post-myocardial infarction tissue repair through affecting apoptosis, cardiac healing, and angiogenesis. Cell Death Dis. 2019;10:480. 31209243 10.1038/s41419-019-1718-7 PMC6579765

[34] Qi G, Zhou Y, Zhang X, et al. Cordycepin promotes browning of white adipose tissue through an AMP-activated protein kinase (AMPK)-dependent pathway. Acta Pharm Sin B. 2019;9:135–143. 30766785 10.1016/j.apsb.2018.10.004 PMC6361849

[35] Fan D, Yang Z, Yuan Y, et al. Sesamin prevents apoptosis and inflammation after experimental myocardial infarction by JNK and NF-kappaB pathways. Food Funct. 2017;8:2875–2885. 28726929 10.1039/c7fo00204a

[36] Zhang X, Hu C, Yuan YP, et al. Endothelial ERG alleviates cardiac fibrosis via blocking endothelin-1-dependent paracrine mechanism. Cell Biol Toxicol. 2021;37:873–890. 33469864 10.1007/s10565-021-09581-5

[37] Zhan W, Liao X, Chen Z, et al. LINC00858 promotes colorectal cancer by sponging miR-4766-5p to regulate PAK2. Cell Biol Toxicol. 2020;36:333–347. 31902050 10.1007/s10565-019-09506-3

[38] Zhang X, Zhu JX, Ma ZG, et al. Rosmarinic acid alleviates cardiomyocyte apoptosis via cardiac fibroblast in doxorubicin-induced cardiotoxicity. Int J Biol Sci. 2019;15:556–567. 30745842 10.7150/ijbs.29907 PMC6367577

[39] Lv LL, Feng Y, Wu M, et al. Exosomal miRNA-19b-3p of tubular epithelial cells promotes M1 macrophage activation in kidney injury. Cell Death Differ. 2020;27:210–226. 31097789 10.1038/s41418-019-0349-y PMC7206053

[40] Zhang X, Hu C, Zhang N, et al. Matrine attenuates pathological cardiac fibrosis via RPS5/p38 in mice. Acta Pharmacol Sin. 2021;42:573–584. 32694761 10.1038/s41401-020-0473-8 PMC8115053

[41] Hu C, Zhang X, Zhang N, et al. Osteocrin attenuates inflammation, oxidative stress, apoptosis, and cardiac dysfunction in doxorubicin-induced cardiotoxicity. Clin Transl Med. 2020;10:e124. 32618439 10.1002/ctm2.124 PMC7418805

[42] Huang SH, Xu M, Wu HM, et al. Isoquercitrin attenuated cardiac dysfunction via AMPKalpha-dependent pathways in LPS-treated mice. Mol Nutr Food Res. 2018;62:e1800955. 30359483 10.1002/mnfr.201800955

[43] Wang SF, Liu X, Ding MY, et al. 2-O-beta-d-glucopyranosyl-l-ascorbic acid, a novel vitamin C derivative from Lycium barbarum, prevents oxidative stress. Redox Biol. 2019;24:101173. 30903981 10.1016/j.redox.2019.101173 PMC6430735

[44] Zhang X, Zhang P, An L, et al. Miltirone induces cell death in hepatocellular carcinoma cell through GSDME-dependent pyroptosis. Acta Pharm Sin B. 2020;10:1397–1413. 32963939 10.1016/j.apsb.2020.06.015 PMC7488361

[45] Luo Z, Kuang XP, Zhou QQ, et al. Inhibitory effects of baicalein against herpes simplex virus type 1. Acta Pharm Sin B. 2020;10:2323–2338. 33354504 10.1016/j.apsb.2020.06.008 PMC7745058

[46] Pan Y, Xie Z, Cen S, et al. Long noncoding RNA repressor of adipogenesis negatively regulates the adipogenic differentiation of mesenchymal stem cells through the hnRNP -PTX3-ERK axis. Clin Transl Med. 2020;10:e227. 33252864 10.1002/ctm2.227 PMC7648959

[47] Wang D, Ruan X, Liu X, et al. SUMOylation of PUM2 promotes the vasculogenic mimicry of glioma cells via regulating CEBPD. Clin Transl Med. 2020;10:e168. 32997416 10.1002/ctm2.168 PMC7507322

[48] Chen L, Chen L, Qin Z, et al. Upregulation of miR-489-3p and miR-630 inhibits oxaliplatin uptake in renal cell carcinoma by targeting OCT2. Acta Pharm Sin B. 2019;9:1008–1020. 31649850 10.1016/j.apsb.2019.01.002 PMC6804444

[49] Canivet CM, Bonnafous S, Rousseau D, et al. Hepatic FNDC5 is a potential local protective factor against non-alcoholic fatty liver. Biochim Biophys Acta Mol Basis Dis. 2020;1866:165705. 32001301 10.1016/j.bbadis.2020.165705

[50] Liu TY, Xiong XQ, Ren XS, et al. FNDC5 alleviates hepatosteatosis by restoring AMPK/mTOR-mediated autophagy, fatty acid oxidation, and lipogenesis in mice. Diabetes. 2016;65:3262–3275. 27504012 10.2337/db16-0356

[51] Huang DQ, El-Serag HB, Loomba R. Global epidemiology of NAFLD-related HCC: trends, predictions, risk factors and prevention. Nat Rev Gastroenterol Hepatol. 2021;18:223–238. 33349658 10.1038/s41575-020-00381-6 PMC8016738

[52] Zhou F, Zhou J, Wang W, et al. Unexpected rapid increase in the burden of NAFLD in China from 2008 to 2018: a systematic review and meta-analysis. Hepatology. 2019;70:1119–1133. 31070259 10.1002/hep.30702

[53] Liu Y, Xu W, Zhai T, et al. Silibinin ameliorates hepatic lipid accumulation and oxidative stress in mice with non-alcoholic steatohepatitis by regulating CFLAR-JNK pathway. Acta Pharm Sin B. 2019;9:745–757. 31384535 10.1016/j.apsb.2019.02.006 PMC6664044

[54] Podszun MC, Alawad AS, Lingala S, et al. Vitamin E treatment in NAFLD patients demonstrates that oxidative stress drives steatosis through upregulation of de-novo lipogenesis. Redox Biol. 2020;37:101710. 32920226 10.1016/j.redox.2020.101710 PMC7494510

[55] Maya-Miles D, Ampuero J, Gallego-Duran R, et al. Management of NAFLD patients with advanced fibrosis. Liver Int. 2021;41(Suppl 1):95–104. 34155801 10.1111/liv.14847

[56] Gaul S, Leszczynska A, Alegre F, et al. Hepatocyte pyroptosis and release of inflammasome particles induce stellate cell activation and liver fibrosis. J Hepatol. 2021;74:156–167. 32763266 10.1016/j.jhep.2020.07.041 PMC7749849

[57] Bostrom P, Wu J, Jedrychowski MP, et al. A PGC1-alpha-dependent myokine that drives brown-fat-like development of white fat and thermogenesis. Nature. 2012;481:463–468. 22237023 10.1038/nature10777 PMC3522098

[58] Zhang X, Hu C, Yuan YP, et al. A brief overview about the physiology of fibronectin type III domain-containing 5. Cell Signal. 2020;76:109805. 33031934 10.1016/j.cellsig.2020.109805

[59] Zhang X, Hu C, Wu HM, et al. Fibronectin type III domain-containing 5 in cardiovascular and metabolic diseases: a promising biomarker and therapeutic target. Acta Pharmacol Sin. 2021;42:1390–1400 . 33214697 10.1038/s41401-020-00557-5 PMC8379181

[60] Zhang HJ, Zhang XF, Ma ZM, et al. Irisin is inversely associated with intrahepatic triglyceride contents in obese adults. J Hepatol. 2013;59:557–562. 23665283 10.1016/j.jhep.2013.04.030

[61] Zhang X, Hu C, Kong CY, et al. FNDC5 alleviates oxidative stress and cardiomyocyte apoptosis in doxorubicin-induced cardiotoxicity via activating AKT. Cell Death Differ. 2020;27:540–555. 31209361 10.1038/s41418-019-0372-z PMC7206111

[62] Bi J, Zhang J, Ren Y, et al. Irisin alleviates liver ischemia-reperfusion injury by inhibiting excessive mitochondrial fission, promoting mitochondrial biogenesis and decreasing oxidative stress. Redox Biol. 2019;20:296–306. 30388684 10.1016/j.redox.2018.10.019 PMC6216086

[63] Polyzos SA, Kountouras J, Anastasilakis AD, et al. Irisin in patients with nonalcoholic fatty liver disease. Metabolism. 2014;63:207–217. 24140091 10.1016/j.metabol.2013.09.013

[64] Monserrat-Mesquida M, Quetglas-Llabres M, Abbate M, et al. Oxidative stress and pro-inflammatory status in patients with non-alcoholic fatty liver disease. Antioxidants (Basel). 2020;9:759. 10.3390/antiox9080759 PMC7463614 32824349

[65] Li DJ, Sun SJ, Fu JT, et al. NAD(+)-boosting therapy alleviates nonalcoholic fatty liver disease via stimulating a novel exerkine Fndc5/irisin. Theranostics. 2021;11:4381–4402. 33754067 10.7150/thno.53652 PMC7977447

[66] Zhou B, Qiu Y, Wu N, et al. FNDC5 attenuates oxidative stress and NLRP3 inflammasome activation in vascular smooth muscle cells via activating the AMPK-SIRT1 signal pathway. Oxid Med Cell Longev. 2020;2020:6384803. 32509148 10.1155/2020/6384803 PMC7254086

