# Supplementary material for: MicroRNA-665-3p exacerbates nonalcoholic fatty liver disease in mice
Source: Bioengineered. 2022 Jan 18;13(2):2927–42. doi: 10.1080/21655979.2021.2017698 (PMC8973643; doi:10.1080/21655979.2021.2017698)
Supplement: Supplemental Material [file KBIE_A_2017698_SM6099.zip › supplementary/Supplementary information.docx]

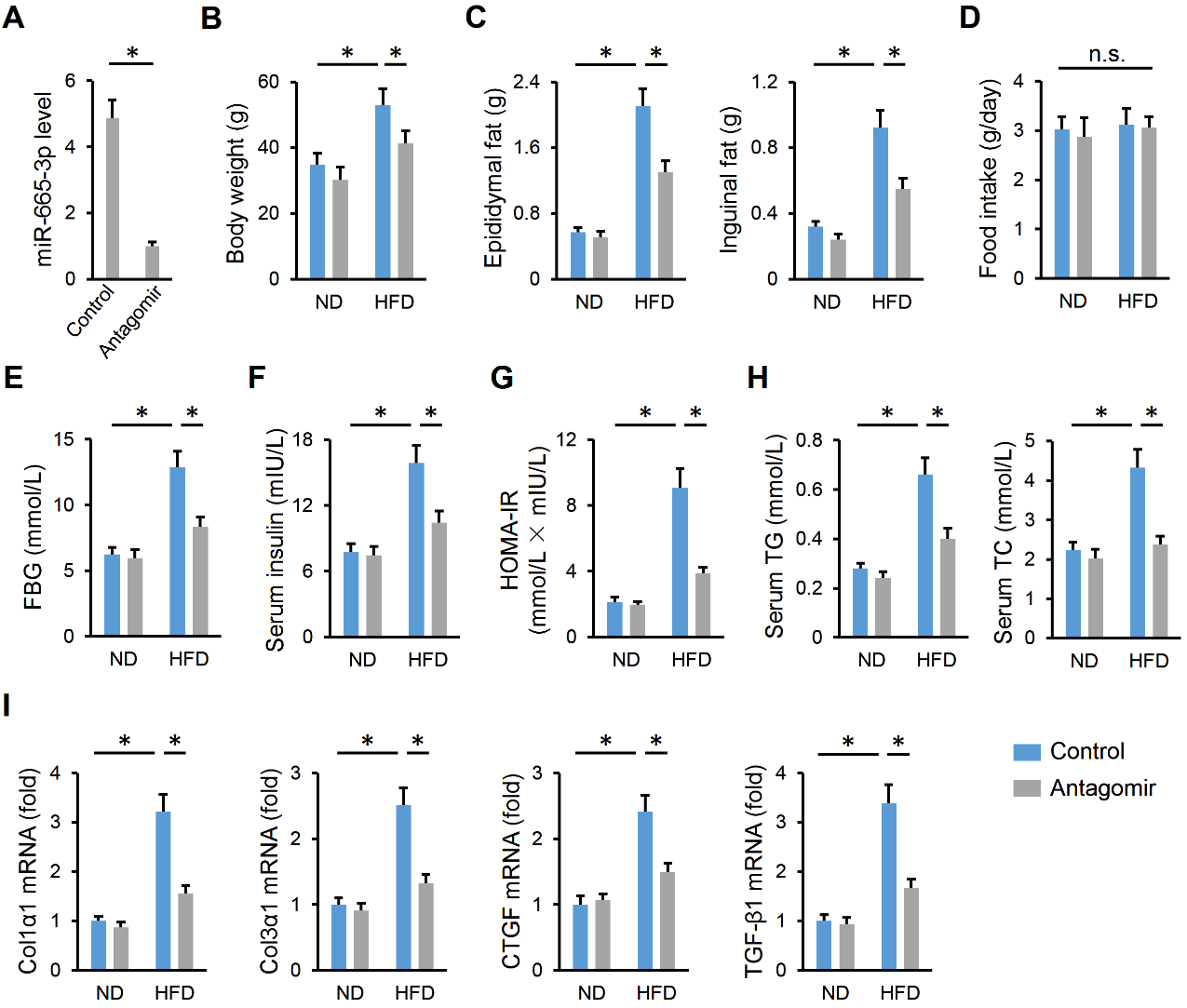


**Figure S1. miR-665-3p antagomir alleviates NAFLD progression in mice.** (A) The levels of miR-665-3p in the liver from mice treated with or without the miR-665-3p antagomir. (B) Body weight. (C) The weights of epididymal and inguinal fat pad. (D) Food intake. (E-F) The levels of FBG and serum insulin in mice. (G) Quantification of the insulin resistance index HOMA-IR. (H) Serum levels of TG and TC in mice. (I) Relative mRNA levels of Col1α1, Col3α1, CTGF and TGF-β1 in the liver. All results were expressed as the means ± standard deviations, n=6 for each group, and **P* < 0.05 was considered statistically significant. n.s. indicated no significance.


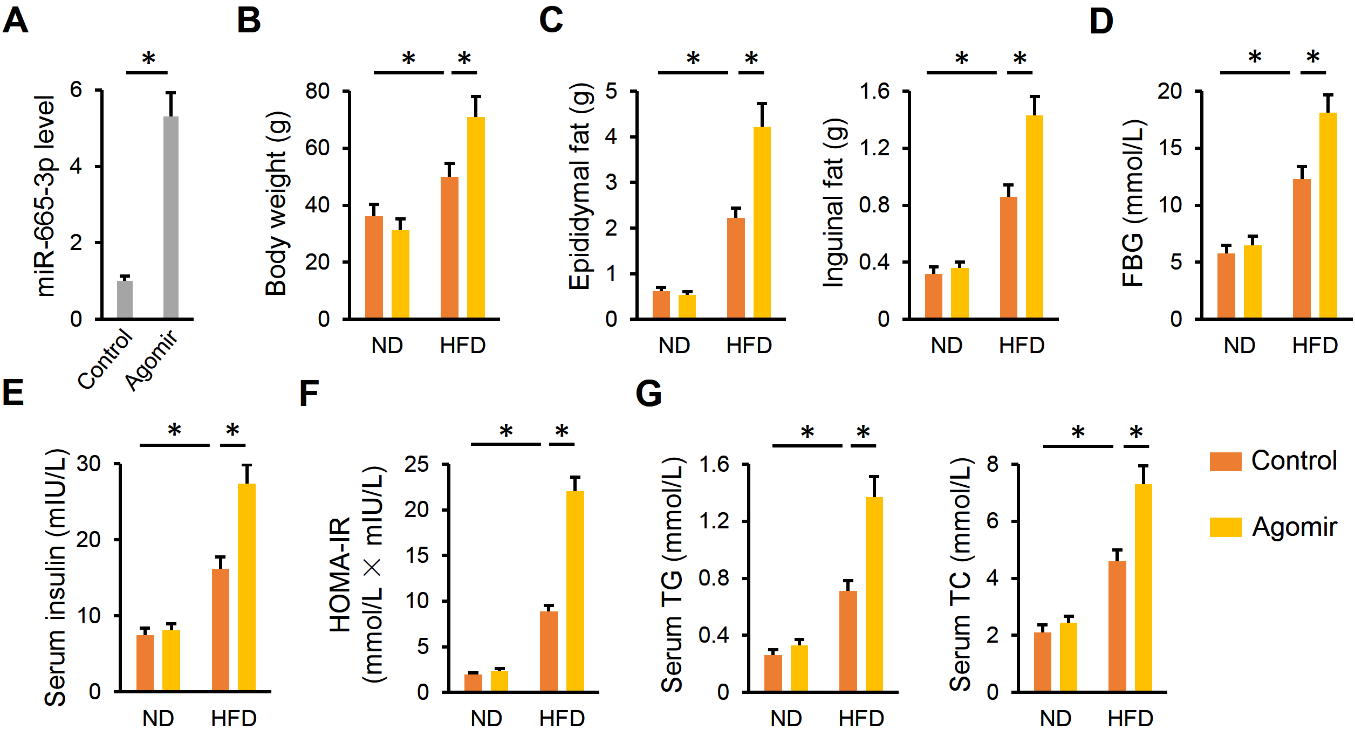


**Figure S2. miR-665-3p agomir facilitates NAFLD progression in mice.** (A) The levels of miR-665-3p in the liver from mice treated with or without the miR-665-3p agomir. (B) Body weight. (C) The weights of epididymal and inguinal fat pad. (D-E) The levels of FBG and serum insulin in mice. (F) Quantification of the insulin resistance index HOMA-IR. (G) Serum levels of TG and TC in mice. All results were expressed as the means ± standard deviations, n=6 for each group, and **P* < 0.05 was considered statistically significant.
